# The Impact of Airway Oxidative Stress on Macrophage Polarization in Stable Asthma and COPD: Association With Clinical Features in a Prospective Observational Study

**DOI:** 10.1111/jcmm.71237

**Published:** 2026-06-08

**Authors:** Magdalena Paplińska‐Goryca, Małgorzata Proboszcz, Monika Wróbel, Magdalena Radziszewska, Katarzyna Mycroft‐Rzeszotarska, Rafał Krenke

**Affiliations:** ^1^ Department of Internal Medicine, Pulmonary Diseases and Allergy Medical University of Warsaw Warsaw Poland

**Keywords:** asthma, COPD, macrophages, oxidative stress, polarization

## Abstract

The polarization status of pulmonary macrophages is dependent on cytokines and mediators present locally in the airways. This study aimed to evaluate whether oxidative stress present in the airways of asthma or COPD patients promotes M1 or M2 macrophage polarization. M1 macrophages predominated in COPD, whereas no increase in M2 macrophages was observed in any of the investigated groups. The elevated number of M1 in COPD was related to a higher level of increased oxidative stress marker mRNA expression, *CYP1B1*, and enhanced IL‐6 and IL‐8 levels. The M2 macrophage polarization was not disease‐related and correlated with higher FEV_1_/VC. Airway oxidative stress is related to M1 macrophage polarization in COPD, associated with increased inflammation and *CYP1B1* mRNA expression. M1 macrophages were not related to clinical features in stable asthma or COPD, whereas higher levels of M2 macrophages were associated with better spirometry results.

## 1 Introduction

Macrophages are the most abundant immune effector cells in the alveolar space. Their role in host defence includes the recognition of pathogen‐associated molecular patterns (PAMPs), damage‐associated molecular patterns (DAMPS), dust particles, and the regulation of inflammatory responses [1]. Induced sputum (IS) is a convenient and valuable respiratory sample rich in macrophages, primarily reflecting the cellular profile of macrophages in the trachea, as well as large and medium‐sized bronchi.

Oxidative stress, in synergy with inflammation, represents the leading pathways of cigarette smoke toxicity and is an important factor in asthma and COPD pathophysiology. Among macrophages, two main subpopulations are distinguished: M1, referred to as the ‘pro‐inflammatory’, and M2, the ‘anti‐inflammatory’ population. Since macrophage polarization is strictly cytokine‐dependent and the profile of mediators secreted in the course of asthma and COPD is complex and variable, it can be challenging to establish a fixed (easily achieved in vitro) macrophage phenotype (which is additionally very plastic) characterizing a specific disease. These variations in macrophage polarization correlate with different clinical outcomes in asthma and COPD [2]. M2‐dominant macrophages are associated with allergic asthma [3], whereas M1‐dominant macrophages are linked to nonallergic inflammation, for example, neutrophilic asthma [4]. It is unknown if oxidative stress is a possible indicator of macrophage polarization and could be one of the factors associated with M1 or M2 macrophage polarization. This study aimed to evaluate whether oxidative stress is related to M1 or M2 macrophage polarization asthma or COPD patients compared to non‐smoking and smoking controls.

## 2 Results and Discussion

**FIGURE 1:**
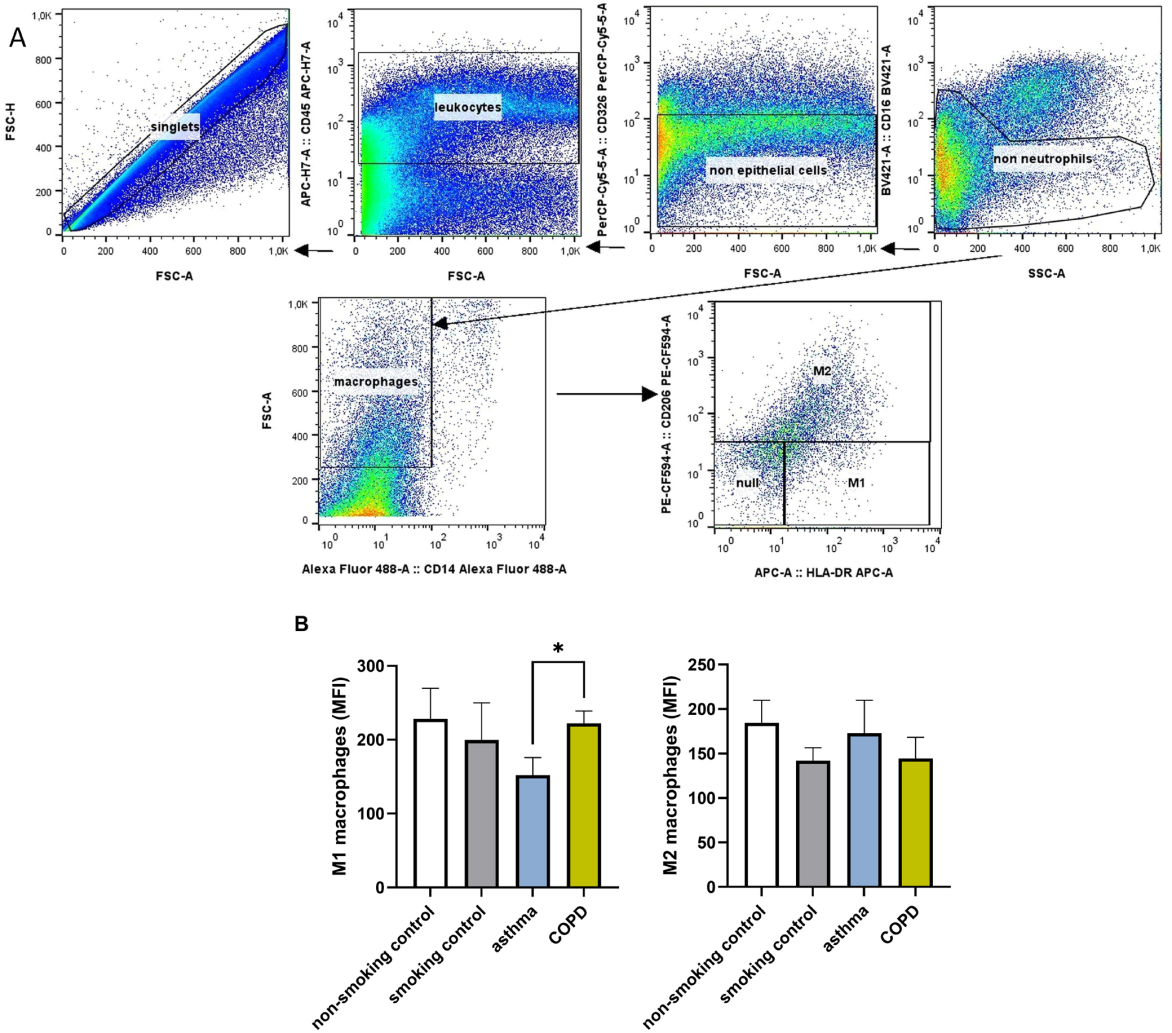
(A) Gating strategy used in this study. After exclusion of dublets, the sputum leukocytes were segregated as CD45+, CD326− cells. In the next step, the fraction of neutrophils was discarded, and the macrophage fraction was characterized as cells with high FSC++ and CD14− expression. The macrophages were identified as CD45^+^CD326^−^CD16^−^CD14^−^FSC^++^cells. Finally, the M1‐like macrophages were characterized as HLA‐DR^++^CD206^−^ macrophages, and M2 as HLA‐DR^+^CD206^+^ cells. (B) The incidence of M1 and M2 macrophage levels in induced sputum of control, asthma, and COPD patients. The data are shown as mean ± SEM. Mann–Whitney test. *p < 0.05.

Macrophages play a vital role in maintaining airway homeostasis. They express a variety of receptors and signalling molecules, and the pathway of their activation and polarization is largely determined by the local microenvironment. Here, we evaluated whether the profile of expression of markers of oxidative stress present in the airways is associated with impaired macrophage biology. In this study, we utilized induced sputum from 54 (15 asthma, 17 COPD, and 22 healthy controls) (14 non‐smokers and 8 smoking controls) Table S2, in which the phenotype of macrophages, mRNA expression of oxidative stress markers, as well as concentration of inflammatory cytokines were measured. The study methodology is described in Appendix S1, Table S1. Analysis of sputum macrophages revealed a higher number of M1 macrophages in the COPD group compared to the asthma group (Figure 1). The level of M2 macrophages did not differ significantly between any of the investigated groups.

**FIGURE 2:**
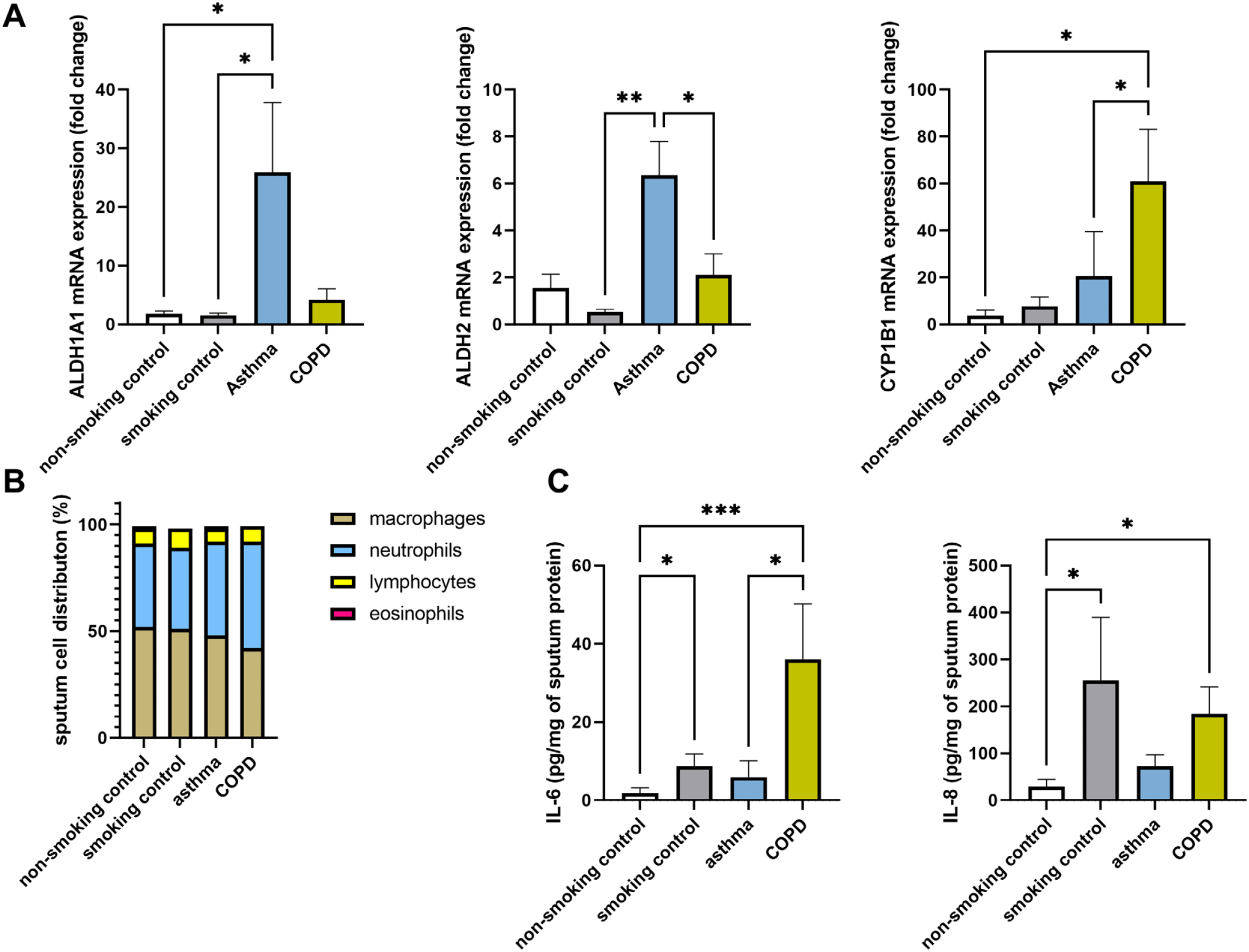
(A) mRNA expression of mediators associated with inflammation and oxidative stress in induced sputum cells of control, asthma, and COPD subjects. (B) The sputum cell distribution among control, asthma, and COPD groups. The sputum cell distribution was similar among the evaluated groups, except for an increased number of neutrophils in the COPD group. (C) The level of IL‐6 and IL‐8 in induced sputum cells of control, asthma, and COPD subjects. The data are shown as mean ± SEM. Data were analysed using the Kruskal‐Wallis test. *p < 0.05, **p < 0.01; ***p < 0.001.

To analyse potential markers associated with inflammation and oxidative stress in asthma and COPD airways, changes in their mRNA expression in the whole sputum cells were measured. PCR analysis revealed increased mRNA expression for *ALDH1A1* in the asthma group compared to non‐smoking and smoking controls. Additionally, *ALDH2* expression was elevated in the asthma group compared to smoking controls and COPD patients (Figure 2). The highest mRNA expression of *CYP1B1* was observed in the COPD. The analysis of protein levels revealed elevated concentrations of IL‐6 and IL‐8 in COPD and in smoking controls, compared to non‐smoking controls. The difference between asthma and COPD was found only for IL‐6. In the whole group, the number of M2 macrophages correlated positively with FEV_1_/VC (*r* = 0.33, *p* = 0.02).

In healthy airways, oxidative stress is maintained at controlled levels, with antioxidant protection primarily mediated by macrophages and epithelial cells [5]. Triggers that can induce uncontrolled pro‐oxidative mechanisms in the airways include infection, aeroallergens, air pollution, cigarette smoke, and inflammation. The imbalance promotes the recruitment of effector immune cells, triggers acute immune responses, and contributes to tissue damage. Macrophages actively respond to oxidative stress through the production of inducible nitric oxide synthase (iNOS), induction of transforming growth factor‐β (TGF‐β) signalling, and activation of NADPH oxidases and mitochondrial ROS (mROS) generation [6]. When the antioxidant–oxidant balance is disrupted, macrophage functions, particularly phagocytic activity, may be impaired, compromising their protective role in the airways.

Published data suggest that oxidative stress might directly impact the metabolism of macrophages, alter cell polarization, as well as affect the M1/M2 balance. During acute tissue injury or bacterial infection, increased accumulation of ROS promotes M1 macrophage polarization and proinflammatory cytokine production, which are key contributors to the inflammatory response [7]. Notably, M1 macrophages exhibit a high tolerance to ROS. Staitieh et al. [8] showed that M1 alveolar macrophages exposed to exogenous oxidative stress via glucose oxidase‐generated hydrogen peroxide more effectively eliminated ROS compared to M2 macrophages. In contrast, M2 macrophages—typically associated with an anti‐inflammatory phenotype—appear to be adversely affected by oxidative stress [9].

The induction of oxidative stress in macrophages involves a complex interplay between mitochondrial injury, endoplasmic reticulum stress, and enhanced M1 polarization under acute stress conditions [10]. Moreover, crosstalk between hypoxia‐related pathways and endoplasmic reticulum stress has emerged as a critical regulator of macrophage polarization [11]. M1 and M2 macrophage polarization displays distinct bioenergetic profiles: M1 macrophage metabolism relies predominantly on enhanced aerobic glycolysis [12], whereas M2 macrophages favour mitochondrial oxidative phosphorylation [13].

It has been shown that cigarette smoke induces M1 polarization via activation of the JNK/c‐Jun pathway [14]. In our study, we observed an increased number of M1 macrophages in the sputum of COPD patients but not in smoking controls. This finding suggests that M1 polarization in COPD airways is associated with a milieu related to increased levels of oxidative stress, as reflected by elevated *CYP1B1* mRNA expression and additional factors such as chronic inflammation marked by upregulated IL‐6 and IL‐8 levels. These associations have also been reported by other authors. *CYP1B1* is a well‐characterized gene implicated in the molecular mechanism of COPD due to its involvement in oxidative stress and xenobiotic metabolism [15]. In contrast, our data indicate that antioxidant defense in asthma may be supported by the activity of aldehyde dehydrogenase (ALDH), which detoxifies aldehydes generated during lipid peroxidation and oxidative stress. Although activation of ALDH is commonly linked to the detoxification of acrolein and aldehydes derived from cigarette smoke, we did not find such a correlation in our study. It is important to note that we measured the expression of only three out of 19 known ALDH isoforms and did not assess enzymatic activity. Therefore, we cannot exclude the possibility of ALDH upregulation in COPD. Nevertheless, our findings suggest that the pattern of ALDH expression and activation differs between asthma and COPD, potentially reflecting disease‐specific oxidative stress triggers.

This study has several limitations. Although bronchoalveolar lavage fluid (BALF) is the gold standard for assessing airway macrophages, its invasiveness led us to use induced sputum—a non‐invasive, macrophage‐rich specimen reflecting bronchial rather than alveolar populations. We propose that oxidative stress in asthma and COPD similarly affects macrophages throughout the respiratory tract, though this requires further validation. In vitro macrophage models could offer greater experimental control but lack the complex disease context essential for understanding macrophage metabolism. The relatively small sample size and inclusion of only patients with stable asthma and COPD limit the generalizability of the findings, particularly to those with acute exacerbations. Finally, the absence of longitudinal and detailed clinical data prevented correlation of molecular results with clinical outcomes, which may partly explain the lack of associations observed between oxidative stress markers and disease severity.

## 3 Conclusions

Airway oxidative processes influence macrophage polarization. Our findings suggest that oxidative stress in COPD is associated with elevated *CYP1B1* mRNA expression and increased inflammatory activity, both of which may promote M1 macrophage polarization. In contrast, M2 polarization did not appear to be disease‐specific. Notably, neither M1 nor M2 macrophage polarization correlated with clinical features in patients with stable asthma or COPD.

## Author Contributions

**Magdalena Paplińska‐Goryca:** supervision, writing – original draft, methodology, investigation, data curation, conceptualization, visualization. **Małgorzata Proboszcz:** methodology, funding acquisition, writing – review and editing. **Monika Wróbel:** writing – review and editing, methodology. **Magdalena Radziszewska:** methodology, writing – review and editing. **Rafał Krenke:** formal analysis, writing – review and editing, validation. **Katarzyna Mycroft‐Rzeszotarska:** methodology, writing – review and editing.

## Funding

This study was funded by an internal grant from the Medical University of Warsaw (Warszawski Uniwersytet Medyczny 1WU/2/M/MB/N/20/20).

## Ethics Statement

All procedures performed in this study were in accordance with the ethical standards of the institutional and/or national research committee and with the 1964 Helsinki declaration and its later amendments or comparable ethical standards. This work has received approval for research ethics from the Medical University of Warsaw Review Board (KB/135/2020) and a proof/certificate of approval is available upon request.

## Conflicts of Interest

R.K. reports personal fees and other from MSD, personal fees and other from AstraZeneca, personal fees from Polpharma, outside the submitted work. The other authors declare no conflicts of interest.

## Supporting information


**Table S1:** Assay ID of primers and TaqMan probes (Thermo Fisher Scientific) used in real‐time qPCR.
**Table S2:** Characteristics of study participants.

## Data Availability

The data that support the findings of this study are available from the corresponding author upon reasonable request.
