# Supplementary material for: The Impact of Airway Oxidative Stress on Macrophage Polarization in Stable Asthma and COPD: Association With Clinical Features in a Prospective Observational Study
Source: J Cell Mol Med. 2026 Jun 8;30(11):e71237. doi: 10.1111/jcmm.71237 (PMC13247113; doi:10.1111/jcmm.71237)
Supplement: Supplementary file 1 — Table S1: Assay ID of primers and TaqMan probes (Thermo Fisher Scientific) used in real‐time qPCR. Table S2: Characteristics of study participants. [file JCMM-30-e71237-s001.docx]

Study design and clinical protocols

This was a prospective, observational, non-randomized study that included asthma and COPD patients, as well as healthy controls. The subjects were recruited from the outpatient clinic at the Department of Internal Medicine, Pulmonary Diseases and Allergy of the Medical University of Warsaw. The study protocol (approved by the Ethics Committee of the Medical University of Warsaw KB/135/2020) included the collection of clinical and demographic data, spirometry, sputum, and blood collection. The size group was calculated according to data from the work of Deshane et al [1], in which the authors, using flow cytometry, characterized immune cells that differed between asthma and COPD. Assuming a mean abundance of M2-expressing macrophages of 31.9±11.5 in asthma and 18.1±12.5 in COPD to detect differences between the study groups with a power of 90% and a significance level of 5%, the group size was estimated for 15 subjects in group.

Patients

Participants were eligible for the COPD group if they met the following criteria: age ≥ 40 years, clinical diagnosis of COPD based on medical history, smoking history of at least 10 pack-years, presence of typical signs and symptoms (shortness of breath especially during physical activity, wheezing, productive chronic cough, chest tightness), and evidence of irreversible airway obstruction confirmed by spirometry defined as a post-bronchodilator FEV1/FVC z-score below -1.645) [2].

Inclusion criteria for asthma patients were as follow: age ≥ 18 years and older, clinical diagnosis of asthma based on medical history, presence if respiratory symptoms (wheezing, shortness of breath, cough, chest tightness), and demonstration of variable expiratory airflow limitation [3].

Patients were excluded if they had a documented current or previous history of asthma (for COPD patients) or a COPD diagnosis (for asthma patients), as well as any other chronic lung disease, autoimmune and haematological diseases, malignancies, severe cardiovascular diseases, chronically used systemic steroids or biologics or if they had experienced a COPD or asthma exacerbation requiring treatment with systemic corticosteroids, and/or antibiotics within the previous 3 months. Spirometry was performed in accordance with the ATS/ERS guidelines [4]. Atopy was diagnosed if at least one skin prick test for inhalant allergens was positive (mean wheal diameter ≥3 mm) or if the specific IgE level for inhalant allergens exceeded 0.35 kU/L.

Control subjects were matched by age and gender to the study group (separate matched non-smoking control group to asthma and smoking control to COPD) and were recruited from volunteers with normal spirometry, no lung conditions, haematological or malignant diseases, and no respiratory tract infections within the previous 3 months. Control subjects were examined by a clinician, and the basal biometric and clinical data were collected in the questionnaire.

Induced sputum collection and processing

Sputum induction was preceded by inhalation of 400 μg of salbutamol. This was followed by inhalation of sterile hypertonic saline (NaCl) solutions at increasing concentrations (3%, 4%, and 5%) using an ultrasonic nebulizer (Tajfun, Medbryt), as recommended by ERS guidelines [5]. IS samples were processed as previously described [6]. Sputum smears were prepared to determine total and differential leukocyte count. The collected sputum cells were used for cytometric and cytological analyses. Supernatants were separated and stored at −80°C for subsequent measurement of cytokine concentrations.

Flow cytometry

BD Pharmingen Human BD Fc Block (BD Biosciences) (5 μl per 100 μl of sample) was added to block non-specific binding. Cells were then stained with antibodies against the following surface binding molecules: CD16 (BV421, mouse anti-human, cat. no. 562874), CD80 (BV510, mouse anti-human cat. no. 567426), CD14 (Alexa Fluor 488, mouse anti-human cat. no. 562689), CD206 (PE-CF594, mouse anti-human cat. no. 564063), HLA-DR (APC, mouse anti-human cat. no. 641402), CD326 (BB700, mouse anti-human cat. no. 745841), CD45 (APC-H7, mouse anti-human cat. no. 641408). The macrophages were identified as CD45^+^CD326^-^CD16^-^CD14^-^FSC^++^cells. The M1-like macrophages were characterised as HLA-DR^++^CD206^-^ macrophages, and M2 as HLA-DR^+^CD206^+^ cells.

Cells were analyzed using a FACSCelesta flow cytometer (BD Biosciences) equipped with blue (488-nm), violet (405-nm), and red (640-nm) lasers. Unstained cells and compensation beads (BD Biosciences) were utilized to set voltages and establish single-stain negative and positive controls. Compensation adjustments were made to address spectral overlap among the seven fluorescent channels used in the study. Samples were initially examined by forward scatter height (FSC-H) versus forward scatter area (FSC-A), followed by side scatter area (SSC-A) versus FSC-A to identify single cells, thus excluding debris and cell clumps from the analysis. Data were processed using FlowJo software version 10.8.1 (Tree Star).

Protein level measurement

Concentrations of inflammatory cytokines/chemokines (IL-12p70, IL-1β, IL-6, IL-8, IL-10, and TNF-α) were determined in cell supernatant using the BD CBA cytometric bead array (BD Bioscience) on a FACSCelesta flow cytometer (BD Biosciences) according to the manufacturer’s instructions. The quantity of the respective cytokine was calculated using CBA software (BD Biosciences). The lower limits of detection of the cytokines are IL-12p70 1.9pg/ml; IL-1β, 7.2 pg/ml; IL-6, 2.5 pg/ml; IL-8, 3.6 pg/ml; IL-10, 3.3 pg/ml; TNF-α, 3.7 pg/ml.

The levels of CCL11 and IL-8 were measured using ELISA (Abcam, Thermo Fisher Scientific) according to the manufacturer’s protocol. The sensitivity of ELISA kits was for CCL11 - 0.41 pg/ml and IL-8 - 2 pg/ml.

Antioxidant status evaluation

Antioxidant capacity was detected using the Antioxidant Assay Kit (Sigma-Aldrich) with Trolox as an antioxidant standard. All measurements were performed according to the manufacturer’s procedures.

mRNA isolation, reverse transcription, and quantitative PCR analysis

Total RNA was extracted from the cells using TRI reagent (Sigma Aldrich). The concentration and purity of the isolated RNA were measured with a DU650 spectrophotometer (Beckman Coulter). cDNA synthesis was performed using 1 μg of total RNA with a cDNA Reverse Transcription Kit and RNase Inhibitor (Thermo Fisher Scientific). cDNA amplification was conducted in an ABI-Prism 7500 Sequence Detector System (Applied Biosystems). For the quantitative PCR (qPCR) reaction, 0.7 µl of cDNA was amplified in a 14 µl PCR volume, which included a TaqMan master mix (Thermo Fisher Scientific) with 150 nM of specific primers for IL-17A, IL-6, IL-8, CCL11, MMP-9, IL-13, IL-4, C15orf48, CYP1B1, CYP1B1-AS1, ALDH2, ALDH3A1 TNF-A, 100 nM of probe (Thermo Fisher Scientific). 18s rRNA was used as a reference gene for normalizing mRNA expression. The assay IDs for the primers and TaqMan probes used in the real-time qPCR are listed in Table S1. Relative quantification values were calculated by the 2^-∆∆CT^ method, and the mean ∆CTs of non-smoking controls were used as calibrators.

Table S1 Assay ID of primers and TaqMan probes (Thermo Fisher Scientific) used in real-time qPCR.

| Gene Symbol | Assay ID |
| --- | --- |
| ALDH1A1 | Hs00946916_m1 |
| ALDH2 | Hs01007998_m1 |
| ALDH3A1 | Hs00964880_m1 |
| C15orf48 | Hs00260902_m1 |
| CCL11 | Hs00237013_m1 |
| CYP1B1 | Hs00164383_m1 |
| CYP1B1-AS1 | Hs00381672_m1 |
| IL-4 | Hs00174122_m1 |
| IL-13 | Hs00174379_m1 |
| IL-17A | Hs00174383_m1 |
| IL-6 | Hs00174131_m1 |
| IL-8 | Hs00174103_m1 |
| MMP9 | Hs00957562_m1 |
| TNF-A | Hs00174128_m1 |
| 18s rRNA | Hs99999901_s1 |

Statistical analysis

Data are presented as mean ± SD for normally distributed data, median and interquartile range ± IQR for non-normally distributed data, or as number and percentage. Differences between nominal variables were evaluated using Pearson’s chi-squared test. Differences between not normally distributed continuous variables across three groups were analysed using Kruskal–Wallis with Dunn’s multiple comparison, or for comparison between two groups with the nonparametric Mann–Whitney U test. Normally distributed samples were analyzed by ANOVA with Tukey’s multiple comparison test. Correlations between variables were analyzed using Spearman’s rank test.

Table S2. Characteristics of study participants.

|  | control  (n=14) | smoking  control  (n=8) | asthma  (n=15) | COPD  (n=17) | p-value |
| --- | --- | --- | --- | --- | --- |
| Age [years] (mean±SD) | 44±14.6* | 52.6±12.6 | 49±12.8# | 64.5±10.6*# | 0.0002 |
| Gender [F/M] (n) | 3/11 | 3/5 | 10/5 | 7/10 | 0.09 |
| BMI (kg/m^2^) (mean±SD) | 31.4±22.4 | 30.1±3.2 | 28.3±5.1 | 28.3±5.1 | 0.88 |
| Smoking status [non-smoker/current smoker/ex-smoker] (n) | 14/0/0 | 0/8/0 | 14/1/0 | 0/11/6 | <0.0001 |
| Smoking exposure [pack-years] (mean±SD) | 0.4±1 *$ | 28.5±15.2$& | 2.5±4.6& | 42.8±28.4* | <0.0001 |
| FEV_1_ [%] (median±IQR) | 96±23.6* | 90±11.5^ | 88±10# | 73±21.5*^# | 0.0002 |
| FVC [%] (median±IQR) | 100±17.2 | 98.5±10.5 | 96±14.7 | 106.6±11 | 0.12 |
| FEV_1_/VC [%] (mean±SD) | 79±8* | 77.2±8.8^ | 74.2±5.9# | 58.5±10.1*^# | <0.0001 |
| Sputum total cell count [x10^6^] (median±IQR) | 2.5±2.9* | 1.8±2.2 | 0.7±1.21 | 0.37±1* | 0.001 |
| Sputum macrophages [%] (median±IQR) | 45±12 | 33±29 | 37.5±39 | 55±10 | 0.07 |
| Sputum lymphocytes [%] (median±IQR) | 7±3 | 3.5±3 | 6.5±6 | 8±6 | 0.08 |
| Sputum neutrophils [%] (median±IQR) | 48±10 | 58±25^ | 52±43 | 34±9^ | 0.01 |
| Sputum eosinophils [%] (median±IQR) | 1±1 | 1±1 | 0±1 | 1±1 | 0.56 |

Data are presented as mean±SD for normally distributed data and median±IQR for not normally distributed data or n. Normally distributed samples were analyzed by ANOVA (p-value) with Tukey’s multiple comparison (symbols), and non-normally distributed data were analyzed by Kruskal-Wallis (p-value) with Dunn’s multiple comparison (symbols) or chi-square test for categorical variables. *Control vs COPD, $ smoking control vs control, ^ smoking control vs COPD, # asthma vs COPD, & smoking control vs asthma.BMI – body mass index, FEV_1_ – forced expiratory volume at first second, FVC – forced vital capacity.

References:

1. Deshane JS, Redden DT, Zeng M, et al (2015) Subsets of airway myeloid-derived regulatory cells distinguish mild asthma from chronic obstructive pulmonary disease. J Allergy Clin Immunol 135:413-424.e15. https://doi.org/10.1016/j.jaci.2014.08.040

2. Quanjer PH, Stanojevic S, Cole TJ, et al (2012) Multi-ethnic reference values for spirometry for the 3-95-yr age range: the global lung function 2012 equations. Eur Respir J 40:1324–1343. https://doi.org/10.1183/09031936.00080312

3. GINA_Report_2010_1.pdf

4. Graham BL, Steenbruggen I, Miller MR, et al (2019) Standardization of Spirometry 2019 Update. An Official American Thoracic Society and European Respiratory Society Technical Statement. Am J Respir Crit Care Med 200:e70–e88. https://doi.org/10.1164/rccm.201908-1590ST

5. Djukanović R, Sterk PJ, Fahy JV, Hargreave FE (2002) Standardised methodology of sputum induction and processing. Eur Respir J Suppl 37:1s–2s. https://doi.org/10.1183/09031936.02.00000102

6. Djukanović R, Sterk PJ, Fahy JV, Hargreave FE (2002) Standardised methodology of sputum induction and processing. Eur Respir J Suppl 37:1s–2s
